# Outbreak of Esophagitis and Ingluvitis Caused by *Salmonella* Typhimurium in Passeriform Birds of the Genus *Sporophila* Seized from Wildlife Trafficking

**DOI:** 10.3390/vetsci11110582

**Published:** 2024-11-19

**Authors:** Karoline L. Soares, Ricardo B. Lucena, Ewerton S. Lima, Millena de O. Firmino, Lilian R. C. Eloy, Raquel Annes F. Silva, Mônica S. Sousa, Isabelle V. Sousa, Weslley Drayton Q. Silva, Artur Cezar de C. Fernandes, Eduardo M. Ramos-Sanchez

**Affiliations:** 1 Graduate Program in Animal Science, Center of Agricultural Sciences, Universidade Federal da Paraiba, Rodovia 12, Areia 58397-000, Paraiba, Brazil; karoline.lacerda@academico.ufpb.br (K.L.S.); ewerton.lima@academico.ufpb.br (E.S.L.); lilian.eloy@academico.ufpb.br (L.R.C.E.); monica.shinneider.sousa@academico.ufpb.br (M.S.S.); ac.carvalhofernandes@gmail.com (A.C.d.C.F.); 2 Productive Unit Coordination, Instituto Federal do Sertão Pernambucano, Campus Floresta, Floresta 56400-000, Pernambuco, Brazil; millena.oliveira@ifsertao-pe.edu.br; 3 Graduate Program in Animal Health and Science, Center for Rural Health and Technology, Universidade Federal de Campina Grande, Avenida Universitária, s/n Bairro Santa Cecília, Patos 58708-110, Paraiba, Brazil; lembre007@gmail.com (R.A.F.S.); isabelle.vieira@academico.ufpb.br (I.V.S.); 4 Veterinary Medicine Course, Center of Agricultural Sciences, Universidade Federal da Paraiba, Rodovia 12, Areia 58397-000, Paraiba, Brazil; weslley.queiroz@academico.ufpb.br; 5 Facultad de Medicina (FAMED), Universidad Nacional Toribio Rodriguez de Mendoza de Amazonas (UNTRM), Chachapoyas 01001, Amazonas, Peru

**Keywords:** avian pathology, histopatology, microbiology, MALDI-TOF mass spectrometry, serotyping, One Health, salmonellosis

## Abstract

The occurrence of esophagitis and ingluvitis caused by *Salmonella* Typhimurium in passerines seized from illegal wildlife trafficking is described. This illegal activity causes stress and leads to lowered immunity in the birds. Additionally, inadequate hygiene conditions predispose the birds to diseases such as salmonellosis. Few studies report the occurrence of *Salmonella*-induced lesions in the esophagus and crop of passerines; therefore, this study aimed to describe the disease in birds of the genus *Sporophila*, as well as to investigate the presence of the bacterium in the environment and determine the antimicrobial resistance profile of the isolated bacteria. Three birds of the genus *Sporophila* were necropsied. In the esophagus and crop, yellowish plaques corresponding to necrosis with bacterial aggregates were observed. *Salmonella* spp. was isolated from these lesions, with genus confirmation via MALDI-TOF mass spectrometry. Environmental samples were collected from the enclosures and cages where the animals were quarantined, and the same bacterium was isolated. In two birds, the serotype S. Typhimurium was identified. Antibiograms performed on the strains from the birds and the environment revealed resistance to antibiotics in the classes of penicillins, sulfonamides, aminoglycosides, monobactams, tetracyclines, and first and third-generation cephalosporins. To the authors’ knowledge, this is the first report of this agent causing death in *Sporophila* due to esophagitis and ingluvitis. It is also the first report of salmonellosis in three species of passerines in Brazil. The study underscores the importance of understanding the pathogens circulating in wild animals, especially within the context of One Health.

## Simple Summary

This study reports cases of esophagitis and ingluvitis caused by *Salmonella* Typhimurium in passerine birds seized from illegal trade, highlighting its relevance to One Health. While few studies document salmonellosis in passerines, this investigation aimed to describe the disease in these species, identify the pathogen in their environment, and assess antimicrobial resistance profiles. Necropsy revealed necrotic lesions in the crop and esophagus, from which *Salmonella* spp. was isolated. The same pathogen was also found in the quarantine environment. Both environmental and animal strains exhibited resistance to multiple antibiotic classes. Serotyping identified the pathogen as the Typhimurium serovar in two birds. This study underscores the need for understanding circulating pathogens in wildlife to develop mitigation strategies, prevent zoonotic transmission, and address antimicrobial resistance.

## 1. Introduction

Most wildlife trafficking victims in Brazil are birds, with this illegal activity impacting around 20% of native bird species, including some that are endangered [1]. Birds of the genus *Sporophila* are significant victims of illegal breeding and trafficking, with many of these seized animals being sent to rehabilitation and screening centers for eventual release back into the wild [2]. Trafficked birds often suffer from poor physical conditions and exhibit liver and biochemical issues due to inadequate nutrition [3]. Furthermore, the stress from trafficking, combined with poor hygiene, leads to weakened immunity, making these birds more susceptible to opportunistic infections and diseases such as salmonellosis [4].

*Salmonella* are Gram-negative bacilli belonging to the Enterobacteriaceae family [5,6]. They are divided into two species, *S. bongori* and *S. enterica* [7]. *S. enterica* is further classified into six subspecies: *S. enterica* subsp. *enterica*, *S. enterica* subsp. *salamae*, *S. enterica* subsp. *arizonae*, *S. enterica* subsp. *diarizonae*, *S. enterica* subsp. *houtenae*, and *S. enterica* subsp. *indica* [5,8]. Based on surface antigen characteristics and biochemical properties, *Salmonella* are subdivided into serovars [6]. Currently, over 2600 serovars have been identified, with 99% of them belonging to *S. enterica* subsp. *enterica* [7,9].

This bacterium can infect humans, domestic animals, wildlife, and even insects, contaminating the environment, water, or food through sick individuals or carriers [4,10,11]. Contamination through the consumption of contaminated eggs and chicken meat makes domestic poultry significant agents in the transmission of salmonellosis to humans [12]. However, recent studies show that wild birds can harbor serovars relevant to the One Health context, acting as vectors for this pathogen to both humans and domestic animals [13,14,15]. Additionally, the circulation of the pathogen in the wild, whether zoonotic or not, can directly impact wild species populations by causing the death of infected individuals [16].

In passerines, the circulation of the bacteria is associated with group feeding practices, especially when humans encourage the congregation of species by providing food in urban and peri-urban environments, or when birds are kept under inadequate conditions in captivity [17]. In these birds, the Typhimurium serovar has been most frequently reported as the cause of outbreaks in Norway [18,19], Sweden [16,20], Switzerland [14], England [21,22,23], Wales [22,23], the United States [24,25,26], Brazil [27], Austria [28], Canada [29], New Zealand [30], Japan [31,32], and Poland [33].

Despite some descriptions, few studies report the occurrence of salmonellosis in free-living passerines [14] or in birds seized from trafficking [3]. Thus, the presence of the pathogen in wild birds may be underdiagnosed, as testing is rarely performed on these birds [17]. Birds that become ill show acute progression and die within 24 h, with reluctance to fly, apathy, and difficulty feeding due to characteristic lesions; necropsy reveals yellowish plaques in the esophagus and crop [14,17]. The aim of this study was to describe the occurrence of salmonellosis in passerines of the *Sporophila* genus seized from wildlife trafficking and kept in a rehabilitation center, describe the presence of the pathogen in the environment, and examine the antimicrobial resistance profile of the isolated strains.

## 2. Materials and Methods

### 2.1. Birds

In 2021, a wildlife screening and rehabilitation center in the state of Paraiba, Brazil, had been experiencing outbreaks of mortality in passerine birds for several weeks. These birds had been rescued a few months earlier and were still undergoing rehabilitation. Over six months, we conducted 101 necropsies on passerines of various species housed in this rehabilitation center. Among these, three birds from the genus *Sporophila* with a history of regurgitation and weight loss were selected for further examination. All these birds had been confiscated and housed in the same building: Bird 1 was a male adult *caboclinho* (*Sporophila bouvreuil*) seized by the environmental police from a residence in the city of João Pessoa; Bird 2 was a male adult *papa-capim* (*Sporophila nigricollis*) seized from a residence in the city of Campina Grande; and Bird 3 was a male adult *golado* (*Sporophila albogularis*) seized from a residence in João Pessoa, along with six other passerine birds. All birds exhibited symptoms of regurgitation and progressive weight loss, ultimately leading to death within two to four weeks after the onset of clinical signs.

### 2.2. Necropsy and Histological Evaluation

Necropsies were performed, and tissue samples were collected from the thoracic and abdominal organs, as well as the brain, skin, and bones. All samples were fixed in 10% buffered formalin for subsequent preparation of histological slides, and then stained with standard hematoxylin and eosin (H&E) staining.

### 2.3. Microbiology

#### 2.3.1. Bird Samples

During necropsy, aseptic samples were collected from lesions in the esophagus and crop of each bird. For microbiological analysis, the samples were cultured on blood agar base enriched with 5% sheep blood and MacConkey agar, then incubated under aerobic conditions at 37 °C. After bacterial growth, identification was performed based on morpho-tinctorial characteristics. For confirmation of the isolate, colonies from the MacConkey agar were subcultured on selective media for the differentiation of *Salmonella* and *Shigella* using Hektoen enteric (HE) agar and *Salmonella-Shigella* (SS) agar, allowing the observation of black colonies that produce hydrogen sulfide (H_2_S) in both media. After identifying the bacterial isolates, they were selected for antimicrobial susceptibility testing using the disk diffusion method on Mueller-Hinton agar. Disks containing standardized concentrations of antimicrobials were applied, followed by incubation for 24 h. After incubation, the inhibition zones were measured to assess resistance to the tested antimicrobials.

#### 2.3.2. Microbiological Environmental Samples

Microbiological samples were collected from three enclosures (Enclosure 1, Enclosure 2, and Enclosure 3) and three cages (Cage 1, Cage 2, and the cage where passeriform birds were quarantined at the wildlife screening and rehabilitation center). To obtain *Salmonella* spp. samples from the enclosures, three sterile drag swabs were prepared—one for each enclosure. Gauze pads were attached to a 70 cm cotton string, packaged, and autoclaved. The drag swab was run across the entire floor of the enclosure, and then placed in a glass tube containing Rappaport–Vassiliadis selective enrichment broth. In the cages, Stuart-type swabs were used—one for each cage—and swabbed across the floor and perches.

The drag swabs were incubated at 37 °C for 24 h, after which the samples were cultured on Hektoen enteric agar and *Salmonella-Shigella* Agar and incubated aerobically at 37 °C, with a reading taken after 24 h. Cultures and isolation from the Stuart-type swabs from the enclosure followed the same protocol used for the bird samples. In samples where *Salmonella* spp. was isolated, another antibiogram was performed in partnership with the Enterobacteria Laboratory at FIOCRUZ.

#### 2.3.3. Antimicrobial Susceptibility Testing

The antimicrobial discs were placed on the surface of the inoculated agar plates, which were then incubated for 24 h at 37 °C. After incubation, the inhibition zone diameters were measured and interpreted according to CLSI M100 (2023) [34]. The isolates were tested for susceptibility to antimicrobials based on their relevance in both veterinary and human medicine. The antimicrobial agents and their respective concentrations were the following: ampicillin (10 µg), amoxicillin (20 µg), clavulanic acid (10 µg), amoxicillin (10 µg), trimetoprim-sulfametoxazol (25 µg, comprising 1.25 µg of trimethoprim and 23.75 µg of sulfamethoxazole comprising 1.25 µg of trimethoprim and 23.75 µg of sulfamethoxazole), trimethoprim (25 µg), sulfamethoxazole (25 µg), chloramphenicol (30 µg), gentamicin (10 µg), streptomycin (10 µg), meropenem (10 µg), ciprofloxacin (5 µg), nalidixic Acid (30 µg), aztreonam (30 µg), tetracycline (30 µg), cefazolin (30 µg), cephalexin (30 µg), cefoxitin (30 µg), ceftriaxone (30 µg), and cefotaxime (30 µg) [35,36].

### 2.4. MALDI-TOF Mass Spectrometry

After being isolated in culture media, the colonies from the samples of the three birds were sent in Brain Heart Infusion (BHI) agar with glycerol and subjected to biochemical confirmation through matrix-assisted laser desorption/ionization—time-of-flight (MALDI-TOF mass spectrometry) analysis, using the ribosomal protein extraction method directly on plate according to Barcelos et al. [37].

### 2.5. Serotyping

Samples in Mueller-Hinton agar were sent to the Enterobacteria Laboratory at FIOCRUZ, Rio de Janeiro, Brazil, where the identification of *Salmonella* spp. serotypes was performed using the slide agglutination method, which evaluates antigenic differences in the cell capsule (capsular antigens “Vi”), the cell wall (somatic antigens “O”), and the flagella (flagellar antigens “H”), as proposed by White—Kauffmann—Le Minor [38].

### 2.6. Polymerase Chain Reaction (PCR) and Bacterial DNA Amplification

The strains sent to the Enterobacteria Laboratory at FIOCRUZ were inoculated on Muller Hinton agar. After microbial growth, DNA extraction was performed, followed by the PCR reactions. *Escherichia coli* K-12 DH5α was used as the negative control for the PCR reactions, while *S.* Enteritidis S.64 and *S.* Typhimurium S.190 prototype strains [39] from the Culture Collection of the National Reference Laboratory for Bacterial Enteroinfections/Enterobacteria Laboratory/IOC/FIOCRUZ were used as positive controls. The primers for the genes under investigation are described in Table 1.

### 2.7. Amplification of Extended Spectrum Beta-Lactamase (ESBL) Genes

Characterization of beta-lactamases was performed using multiplex PCR analysis (Table 2). The *Klebsiella pneumoniae* strain ATCC 700603 was used as a positive control, and the *Esherihia coli* strain ATCC 25922 served as a negative control.

## 3. Results

### 3.1. Macroscopic and Histopathological Lesions

**Figure 1:**
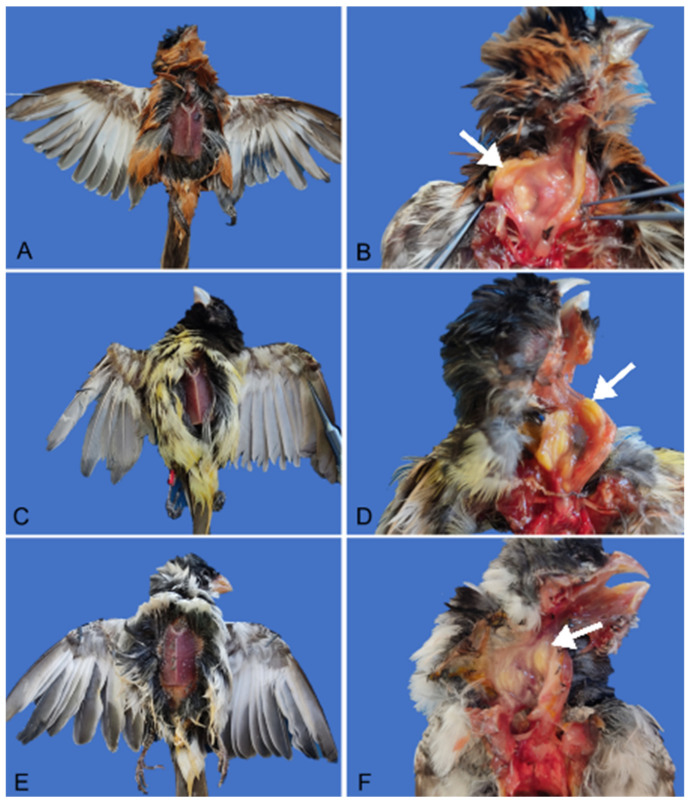
Body score of *Sporophila* genus birds and macroscopic appearance of esophagitis and ingluvitis caused by *Salmonella* Typhimurium. (**A**) Bird 1, male adult *caboclinho* (*Sporophila bouvreuil*), body score of 2 (on a scale of 1–5). (**B**) Bird 1, yellowish plaque 0.4 cm in diameter extending from the serosa to the mucosa of the esophagus and crop. (**C)** Bird 2, male adult *papa-capim* (*Sporophila nigricollis*), body score of 2 (on a scale of 1–5). (**D**) Bird 2, multifocal to coalescent yellowish plaques ranging from 0.1 cm to 0.3 cm in diameter on the mucosa and serosa of esophagus and crop. (**E**) Bird 3, male adult *golado* (*Sporophila albogularis*), body score of 3 (on a scale of 1–5). (**F**) Bird 3, multifocal to coalescent yellowish plaques ranging from 0.2 cm to 0.3 cm in diameter on the mucosa and serosa of the esophagus and crop.

Upon external examination, all birds were found to be underweight, with body scores ranging from 2 to 3 on a scale of 1 to 5. Bird 2 also exhibited amputation of the carpus, metacarpus, and phalanges of the right wing. In the esophagus of all birds, yellowish plaques measuring 0.1 to 0.4 cm in diameter were observed, involving both the serosal and mucosal layers (Figure 1). These plaques extended into the crop in birds 1 and 3. The plaques had an irregular and shiny surface. Upon sectioning, they were soft, compact, and yellowish.

**Figure 2:**
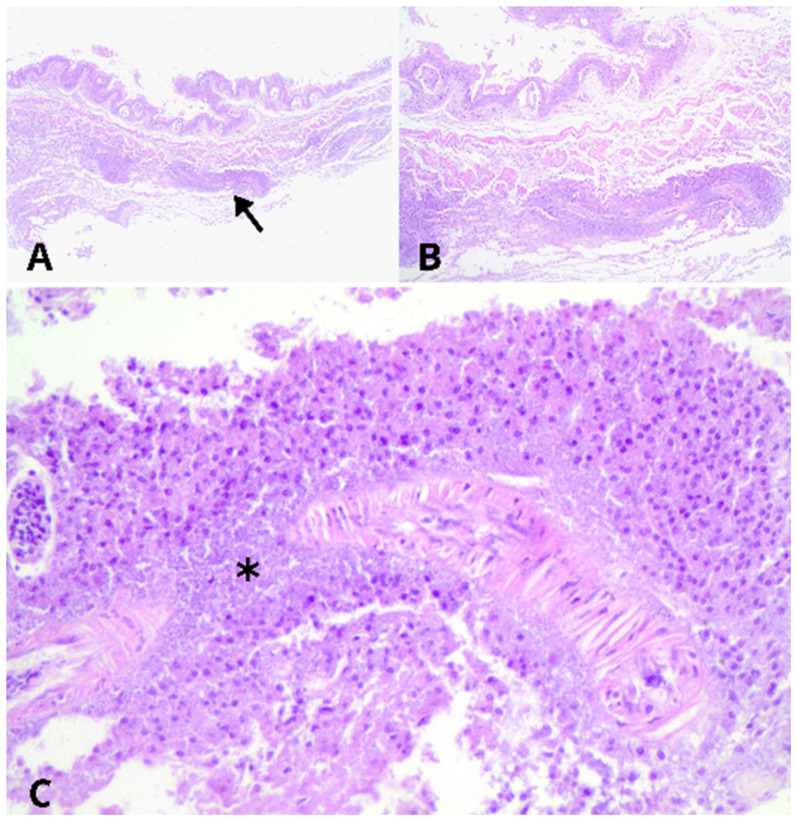
Histopathology of esophagitis caused by *Salmonella* Typhimurium in a *Caboclinho* (*Sporophila bouvreuil*). (**A**–**C**) The crop exhibits multifocal areas of ulceration on the mucosal surface and necrosis in the submucosa (arrow), associated with heterophils, fibrin, and bacterial aggregates (asterisk). Observed under a 4× objective lens (**A**), 10× objective lens (**B**), and 40× objective lens (**C**). All images stained with hematoxylin and eosin.

**Figure 3:**
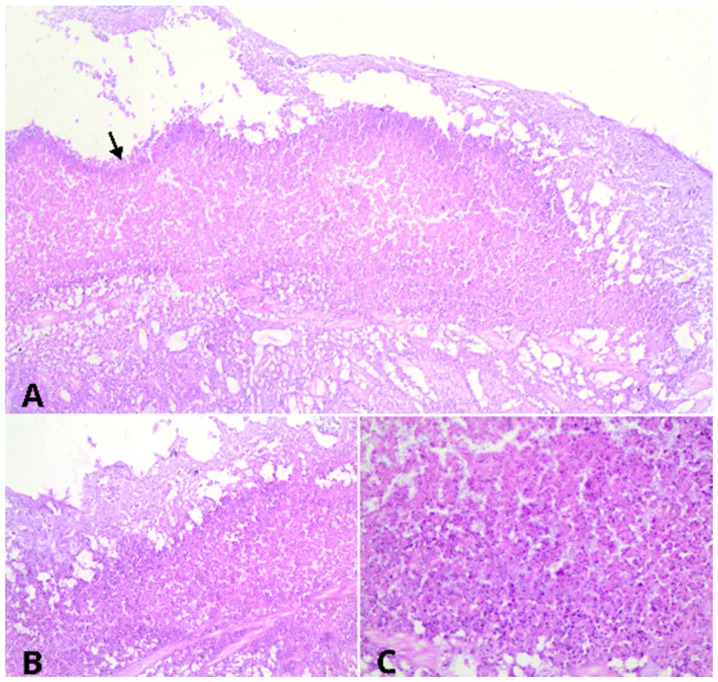
Histopathology of ingluvitis caused by *Salmonella* Typhimurium in a *Papa-capim* (*Sporophila nigricollis*). (**A**–**C**) The crop exhibits an extensive area of ulceration with adjacent necrosis, associated with heterophils, macrophages, fibrin, and bacterial aggregates. Observed under a 4× objective lens (**A**), 10× objective lens (**B**), and 40× objective lens (**C**). All images stained with hematoxylin and eosin.

Histopathological evaluation revealed multifocal to coalescent ulcers of the mucosal surface in the esophagus and crop. Multifocal to coalescent necrosis was observed in the submucosa (Figure 2) or extensive necrosis (Figure 3), accompanied by the presence of heterophils, macrophages, fibrin, and a large number of short rod-shaped bacteria. Numerous blood vessels were occluded by fibrin thrombi, along with a small occasional presence of short rod-shaped bacteria. These lesions extended to the serosa of both organs but were more severe in the crop.

### 3.2. Microbiology and Antibiogram

Bacterial cultures from the birds and the environment, which were plated on Hektoen enteric (HE) agar and *Salmonella-Shigella* Agar (SS), exhibited hydrogen sulfide production, characterized by a dark color at the center of the colonies. On MacConkey agar, the *Salmonella* colonies appeared colorless due to the lack of lactose fermentation. Birds 1, 2, and 3, Enclosure 2, and Cage 2 showed a dark center in the colonies on SS and HE due to hydrogen sulfide production. On MacConkey agar, they were lactose non-fermenters, appearing colorless.

In the antibiogram, samples from three birds, Enclosure 2, and Cage 2 showed resistance to different classes of antibiotics (Table 3).

### 3.3. MALDI-TOF Mass Spectrometry Results

In the mass spectrometry technique, the genus of the strains isolated from the esophageal and ingluvial lesions of birds 1, 2, and 3 was confirmed (Table 4).

### 3.4. Serotyping Results

All strains were identified through serotyping as belonging to the genus *Salmonella*, species *enterica*, and subspecies *enterica*. Bird 1 and Enclosure 2 had the somatic antigens O:4 and O:5 identified. In Birds 1 and 2, it was possible to identify all antigens, allowing for the classification of the Typhimurium serotype in samples from these individuals (Table 5).

### 3.5. Identification of Virulence Genes and Characterization of Extended Spectrum Beta-Lactamase (ESBL) Genes

Variable virulence genes (*orgA*, *invA*, *ssaQ*, *mgtC*, *sopB*, *stn*, and *spvC*) were identified in *Salmonella* strains (Table 6). The molecular investigation of β-lactamase production, performed through polymerase chain reaction (PCR), detected the presence of the bla_CMY_ gene in all five tested *Salmonella* strains.

## 4. Discussion

The confirmation of mortality in passerine birds with lesions in the upper digestive tract associated with *Salmonella* infection in this study was possible through a set of diagnostic techniques. This enabled the elucidation of the pathogenesis of the disease characterized by regurgitation, weight loss, and death in three bird species of the genus *Sporophila*. To the best of the authors’ knowledge, this is the first confirmation of esophagitis and ingluvitis in this bird species. These birds are among the most trafficked wildlife species in Brazil [48], and thus they could be involved in the spread of salmonellosis to other animals or humans.

Our study identified the infection of *Salmonella enterica* subsp. *enterica* serovar Typhimurium in both the birds and the environment where they were being rehabilitated. Birds held in rehabilitation screening centers in Brazil, after a quarantine period, may later be released back into the wild [2]. These birds returning to the wild can contaminate the environment and have impacts on species conservation. Many cases of infection by *Salmonella enterica* subsp. *enterica* serovar Typhimurium have been described in humans associated with the contamination of animal-origin foods, mainly eggs and chicken meat [49,50]. On the other hand, recent studies show that wild birds can harbor serovars relevant to One Health, as they can be vectors of this agent both to people and to domestic animals [13,14,15,51,52,53].

The history of trafficking and interaction with other species may have contributed to the development of fatal diseases in the birds of this study. This differs from reports of isolation of the Typhimurium serovar in passerines in Brazil, where the agent was isolated from cloacal swabs of asymptomatic carrier birds [27]. We still do not know the main serovar found in Brazilian birds, but studies of avian salmonellosis outbreaks in different countries have proven that *Salmonella* Typhimurium is the most prevalent serovar in passerines [54]. Infection in these cases is usually related to the aggregation of species at feeding sites provided by people [13,19], adaptation of birds in urban areas [14], or contact with livestock that may harbor the pathogen [17]. These factors are also observed in trafficked birds in Brazil. In addition to contact with people due to trafficking, birds of the genus *Sporophila* have the habit of feeding on grass seeds and thus may have urban or rural habits [55], which further favors contact with humans and other animals. These factors increase the risk of both contamination and transmission of salmonellosis, since the Typhimurium serovar is considered one of the main causes of disease outbreaks in people, as well as in other animals, such as domestic cats [14,20] due to hunting habits [56]. Additionally, these birds make seasonal long-distance movements (>1000 km) [55], which can result in the dispersion of the agent to other regions.

The isolation of the bacterium in one enclosure and cage at the location where the birds were housed indicates the circulation of the pathogen and its persistence in the environment. This characteristic is due to *Salmonella*’s ability to form biofilms through microbial clusters that can resist on inert or living surfaces [57]. This pathogen has been isolated in enclosures, cages, and waterers at wildlife screening centers, associated with the high demand of animals that can be carriers of this agent [58,59]. This highlights the importance of monitoring salmonellosis in animals at wildlife screening centers that will be reintroduced into the wild.

The virulence genes identified in the *Salmonella* isolates from this study, such as invA, ssaQ, mgtC, and sopB, play crucial roles in the bacterium’s ability to invade and survive within the host. Studies have shown that these genes are associated with various Salmonella pathogenicity islands (SPIs), especially in birds, contributing significantly to the pathogen’s ability to cause infections [60,61].

The invA gene is particularly important for its role in enabling the bacterium to invade intestinal epithelial cells, which is a key step in initiating infection, while ssaQ and sopB assist in the secretion of virulence proteins that aid bacterial survival within host cells [60]. Additionally, the stn gene is responsible for enterotoxin production, which contributes to the inflammatory response in the host [62], and spvC is part of a plasmid virulence operon that helps Salmonella evade the host’s immune defenses, thus promoting the spread of infection [63]. Therefore, the presence of genes like stn and spvC in these isolates highlights *Salmonella*’s enhanced ability to cause disease and underscores the need for monitoring Salmonella in wild bird populations, especially in rehabilitation centers, where cross-species transmission risks are higher.

Although it was not possible to detect all somatic and flagellar antigens of Bird 1 and Enclosure 2, the presence of O:4 and O:5 in these bacteria indicates they belong to serovars of group B, which may or may not be the Typhimurium serovar found in Birds 2 and 3. In Cage 2, it was possible to identify the presence of *Salmonella enterica* subsp. *enterica* without determination of the serovar. *Salmonella* serotypes are defined from an antigenic formula that considers somatic, flagellar, and capsular antigens. The “O” somatic antigens are also called major antigens and divide the bacteria into several serogroups; serogroup B includes strains possessing the O:1, O:5, O:12, and O:27 antigens, among which are the Paratyphi B and Typhimurium serovars [64]. However, due to the phenotypic differentiation of isolates from a specific bird and its corresponding cage, we cannot rule out the possibility of contamination of the cages and surrounding environment by bacteria shed by other birds in the enclosure. This contamination may have resulted from infected birds spreading *Salmonella* to the shared environment, making it challenging to distinguish the primary source of infection.

Multi-drug resistance was observed in the *Salmonella* isolates from the three birds, one enclosure, and the cage, both in the evaluation conducted at UFPB and at Fiocruz. Resistance was demonstrated to various classes of antibiotics, including ampicillin, amoxicillin-clavulanate, amoxicillin, streptomycin, and tetracycline. This pattern is consistent with previous research that identified resistance in *Salmonella* isolates from wild birds in Brazil. For example, studies have shown that wild birds in areas with high human interaction can act as asymptomatic carriers of resistant strains of *Salmonella* spp., posing a significant risk to public health and food safety [65,66].

Streptomycin, an aminoglycoside antibiotic, is not typically used for treating salmonellosis but is commonly used as a growth promoter in animals, potentially leading to the emergence of resistant isolates throughout the production chain. Drug-resistant strains may contaminate not only meat but also soil and vegetation that could be ingested by other species [67]. Resistance mechanisms to tetracycline have also been linked to its use in livestock and its environmental discharge [67]. All samples in this study were resistant to both streptomycin and tetracycline.

Beta-lactam resistance in this study was observed in derivatives of penicillin, cephalosporins, carbapenems, and monobactams. This resistance can be attributed to multiple mechanisms, including the production of beta-lactamase enzymes by certain strains of Enterobacteriaceae, which can inactivate specific antibiotics [68]. However, not all resistant strains exhibit this enzyme production. Additionally, modifications in penicillin-binding proteins (PBPs) and decreased permeability of the bacterial cell membrane may also play significant roles in beta-lactam resistance [69].

On the other hand, sulfonamide resistance occurs through the inhibition of dihydropteroate synthase, an enzyme involved in bacterial DNA and RNA synthesis. This resistance is frequently linked to the presence of genes that confer resistance to the drug [70].

Generally, the isolates from both the birds and the environment exhibited a resistance profile similar to that reported for S. Typhimurium. An evaluation of 11,447 strains showed the tetra-resistant ASSuT pattern (ampicillin, streptomycin, sulfonamides, tetracycline) and the penta-resistant ACSSuT pattern (ampicillin, chloramphenicol, streptomycin, sulfonamides, tetracycline) [49], similar to the resistance patterns found in this study on a smaller scale.

In addition to the microbiological findings, the esophageal nodules observed in the crop and esophagus of the three birds are considered pathognomonic lesions of salmonellosis in passerines associated with the Typhimurium serovar [13,17]. Similar lesions have been observed in outbreaks of salmonellosis in *Carduelis spinus* in Austria [28] and Switzerland [14], in *Pyrrhula pyrrhula* and three species of Carduelis in Norway [19], *Passer montanus* in Japan [31], as well as *Carpodacus purpureus*, *Coccothraustes vespertinus*, and three species of the genus *Carduelis* in Canada [29]. These organs are the initial site of infection. After the development of these masses, sepsis occurs without intestinal lesions [28]. The reason for the development of lesions in this region is not yet known, but it is believed that there is an affinity for this tissue by the bacteria, associated with the way of contamination through the ingestion of food contaminated by feces [21]. Microscopic lesions are characterized by transmural ulcerative ingluvitis and esophagitis to varying degrees, infiltration of viable and degenerated histiocytes and heterophils, with the presence of intra and extracellular bacterial rods surrounding the necrotic areas and expanding the esophageal wall [14,19].

Death occurs due to septic shock favored by wasting and difficulty of the bird in feeding due to esophageal obstruction caused by the masses. The strains of this serovar seem to be adapted to the groups of birds they can affect, thus they can cause disease with high mortality rates in some groups and others being only asymptomatic carriers [71], which may explain the occurrence of death in animals of the same order in this description. To the authors’ knowledge, until now, there had been no report of this esophageal form of salmonellosis in passerines in Brazil.

## 5. Conclusions

The reported cases in this study highlight the harmful potential of *Salmonella* Typhimurium in passerines of the *Sporophila* genus and underscore the importance of curbing wildlife trafficking, as it was a significant factor in the development of the disease in these species. Furthermore, knowledge of the pathogens circulating in wild animals enables the development of mitigative measures to prevent the loss of individuals and protect against zoonoses, since wild animals can act as vectors or reservoirs of diseases for humans and other animals.

## Figures and Tables

**Table 1:** Primers used for the identification of virulence genes of *Salmonella* Typhimurium causing esophagitis and ingluvitis in birds of the genus *Sporophila*.

Gene	Oligonucleotide Sequence (5′-3′)	bp	Reference
*sefA*	F: AGGTTCAGGCAGCGGTTACT	312	[40]
	R: GGGACATTTAGCGTTTCTTG		
*invA*	F: TGCCTACAAGCATGAAATGG	500	[41]
	R: AAACTGGACCACGGTACAA		
*slyA*	F: GCCAAAACTGAAGCTACAGGTG	700	
	R: GTATCGACCACCACGATGGTT		
*phoP/Q*	F: ATGCAAAGCCCGACCATGACG	299	
	R: GTATCGACCACCACGATGGTT		
*orgA*	F: GATAAGGCGAAATCGTCAAATG	540	[42]
	R: GTAAGGCCAGTAGCAAAATTG		
*mgtC*	F: TGACTATCAATGCTCCAGTGAAT	655	
	R: ATTTACTGGCCGCTATGCTGTTG		
*spv*C	F: ACTCCTTGCACAACCAAATGCGGA	424	
	R: TGTCTTCTGCATTTCGCCACCATCA		
*stn*	F: TTAGGTTGATGCTTATGATGGACACCC	617	
	R: CGTGATGAATAAAGATACTCATAGG		
*ssaQ*	F: GAATAGCGAATGAAGAGCGTCC	677	
	R: CATCGTGTTATCCTCTGTCAGC		
*sopB*	F: GATGTGATTAATGAAGAAATGCC	1170	[43]
	R: GCAAACCATAAAAACTACACTCA		
*HilA*	F:GGATCAGGTTCAATCCGAGA	500	
	R: AGTAAGGCGCAATGCTGTTT		
*sipA*	F: CAAACGTTGATACCCCTGCT	766	
	R: CGGTCGTACCGGCTTTATTA		
*Sii*E	F: GCGCAAAAGTTTCTCTTTCCGGG	608	[44]
	R:TTTCTGGTTAGTTATCGGCAGAGTAAACTCTTCT		
*sse*A	F: TTCACCAAATCCGGGCTA	135	[45]
	R: TCTCGGCCTCCTGGTTAA		
*Ssr*B	F: CTTAGTCTACCTGGCATCAATGGC	177	
	R: CGCTAACAGAACTTGCTGACTACTGC		

F: forward primer; R: reverse primer; pb: base pair.

**Table 2:** Primers to identify extended spectrum beta-lactamase (ESBL) genes in *Salmonella* Typhimurium causing esophagitis and ingluvitis in birds of the genus *Sporophila*.

Gene	Oligonucleotide Sequence (5′-3′)	bp	Reference
*bla_TEM_*	F: CATTTCCGTGTCGCCCTTATTC	800	[39,46]
	R: CGTTCATCCATAGTTGCCTGAC		
*bla_SHV_*	F: AGCCGCTTGAGCAAATTAAAC	713	
	R: ATCCCGCAGATAAATCACCAC		
*bla_OXA_*	F: GGCACCAGATTCAACTTTCAAG	564	
	R: GACCCCAAGTTTCCTGTAAGTG		
*bla_CTX-M-1_*	F: CGTTAACGGCACGATGAC	688	
	R: CGATATCGTTGGTGGTRCCAT		
*bla_CTX-M-2_*	F: TTAGGTTGATGCTTATGATGGACACCC	404	
	R: CGATATCGTTGGTGGTRCCAT		
*bla_CTX-M-9_*	F: TCAAGCCTGCCGATCTGGT	561	
	R: TGATTCTCGCCGCTGAAG		
*bla_CTX-M8/25_*	F: AACRCRCAGACGCTCTAC	326	
	F: TCGAGCCGGAASGTGTYAT		
*bla*_*CMY-2*_	R: GCACTTAGCCACCTATACGGCAG	920	[47]
	F: GCTTTTCAAGAATGCGCCAGG		

F: forward primer; R: reverse primer; pb: base pair.

**Table 3:** Antibiogram of bacterial cultures from birds of the genus *Sporophila*, necropsied with esophagitis and ingluvitis lesions caused by *Salmonella* Typhimurium, as well as cultures from the enclosures and cages where these birds were quarantined.

Class Antibiotic		Bird 1	Bird 2	Bird 3	Encl. 2	Cag. 2
Ampicillin		R	R	R	R	R
Amoxicillin + Clavulanate		R	R	R	R	R
Amoxicillin		R	R	R	R	R
Sulfonamides	Trimethoprim-sulfamethoxazole	S	S	R	S	S
	Trimethoprim	S	S	R	S	S
	Sulfamethoxazole	S	S	S	S	S
Phenicoles Chloramphenicol		S	S	S	S	S
Gentamicin		S	S	S	S	S
Streptomycin		R	R	R	R	R
Carbapenems Meropenem		S	S	S	S	S
Nalidixic Acid		S	S	S	S	S
Ciprofloxacin		S	S	S	S	S
Monobactams Aztreonam		R	S	S	S	S
Tetracycline Tetracycline		R	R	R	R	R
Cefazolin		S	S	S	R	R
Cephalosporins 1st						
Cephalexin		R	R	S	S	S
Cephalosporins 2st Cefoxitin		S	S	S	S	S
Ceftriaxone		S	S	S	S	S
Cefotaxime		R	S	S	S	S

**Table 4:** Results of the matrix-assisted laser desorption ionization-time of flight (MALDI-TOF mass spectrometry) technique on bacterial cultures from birds of the genus Sporophila, necropsied with esophagitis and ingluvitis lesions caused by Salmonella Typhimurium.

	Bird 1	Bird 2	Bird 3
Identified Bacterium	*Salmonella* sp. *Enterococcus faecalis*	*Salmonella* sp.	*Salmonella* sp.
Bird 1: *Sporophila bouvreuil*; Bird 2: *Sporophila nigricollis*; Bird 3: *Sporophila albogularis*.			

**Table 5:** Serotyping of bacterial cultures from birds of the genus *Sporophila*, necropsied with lesions of esophagitis and ingluvitis caused by *Salmonella* Typhimurium, as well as cultures from the enclosures and cages where these birds were quarantined.

Sample	Serotyping
Bird 1	*Salmonella enterica* subesp. *enterica* (O:4,5) *
Bird 2	*Salmonella* ser. Typhimurium
Bird 3	*Salmonella* ser. Typhimurium
Cage 2	*Salmonella enterica* subesp. *enterica* *
Enclosure 2	*Salmonella enterica* subesp. *enterica* (O:4,5) *

*: Flagellar structure not identifiable.

**Table 6:** Virulence genes in bacterial cultures from birds of the genus *Sporophila*, necropsied with lesions of esophagitis and ingluvitis caused by *Salmonella* Typhimurium, as well as cultures from the enclosure and cage where these birds were quarantined.

Sample	Genes
Bird 1	*orgA*, *inA*, ssaQ, *mgtC*, *sopB*, *stn*, spvC
Bird 2	*orgA*, *inA*, ssaQ, *mgtC*, *sopB*, *stn*, spvC
Bird 3	*orgA*, *inA*, ssaQ, *mgtC*, *sopB*, *stn*, spvC
Cage 2	*orgA*, *inA*, ssaQ, *mgtC*, *sopB*, *stn*, spvC
Enclosure 2	*orgA*, *inA*, ssaQ, *mgtC*, *sopB*, *stn*, spvC

## Data Availability

The original contributions presented in this study are included in the article. Further inquiries can be directed to the corresponding authors.
